# Health Technology and the Role of Human and Organisational Factors in Planetary Health: a Rapid Integrative Review of Current Literature

**DOI:** 10.1055/s-0046-1816625

**Published:** 2026-11-03

**Authors:** Vaibhav Tyagi, Kirsten Jackson, Melissa Baysari

**Affiliations:** 1 Susan Wakil School of Nursing and Midwifery, Faculty of Medicine and Health, The University of SydneyAustralia; 2 Sydney Environment Institute, The University of SydneyAustralia

**Keywords:** Planetary Health, Human and Organisational Factors, Digital Health, Health Information Technology, Climate Change, Environmental Health

## Abstract

**Objective** : Climate change and environmental degradation pose significant challenges to human health and animal habitation, with healthcare contributing nearly 5% of global carbon emissions. Health technologies, such as telemedicine and electronic health records, offer potential environmental benefits by reducing travel and optimizing resource use. However, health technology impact is complex, and concerns have been raised about these technologies resulting in increased energy consumption, e-waste, and resource depletion.

**Methods** : This rapid integrative review explored the relationship between health technology and planetary health, and examined whether human and organizational factors (HOFs) were considered in this relationship. A structured literature search identified 14 studies examining the environmental impact of health technologies. Eleven of these studies focused on estimating carbon emission reductions due to decreased patient travel, primarily in the United States. No studies examined HOFs, despite the important role HOFs, such as institutional policies, technology design, or user behaviours play in influencing the environmental impact of health technologies.

**Results** : We recommend that future research adopts a systems-based approach, incorporating diverse methodologies to assess real-world impacts. Additionally, understanding how HOFs affect technology adoption and sustainability is crucial for maximizing planetary health benefits. This review showed that while digital health technologies can reduce healthcare-related carbon footprints, their overall environmental impact remains underexplored.

**Conclusion** : Addressing research gaps and integrating HOFs into future studies will be essential for designing and implementing sustainable healthcare solutions that align with planetary health goals.

## 1. Introduction

Climate change and environmental degradation are among the most pressing current global challenges, with significant implications for human health and healthcare systems. The concept of planetary health, which recognizes the interdependence of human health and the natural ecosystems that support it, has gained increased attention in research and policy [ 1 ], acknowledging the way health impacts the environment, and the environment impacts human health. Healthcare is a substantial contributor to environmental degradation, accounting for nearly 5% of global carbon emissions through energy-intensive practices, medical waste, and patient travel [ 2 ]. Health technologies, such as telemedicine, remote patient monitoring, and electronic health records, have been proposed as potential solutions for mitigating some of healthcare's environmental footprint by reducing travel, optimizing resource use, and enhancing efficiency [ 3 4 ]. However, the impact of health technologies on planetary health is complex and multifaceted. For example, while virtual care models have been shown to reduce carbon emissions associated with patient transport [ 5 ], they also introduce environmental costs, including increased energy consumption for digital infrastructure, e-waste production, and resource depletion linked to device manufacturing [ 6 ].

There is now little doubt that the adoption and effectiveness of health technologies are heavily influenced by human and organizational factors (HOFs) [ 7 8 9 10 ]. Human factors, the discipline concerned with understanding and improving work, tells us that the interactions between people and technologies are influenced by technology design and the capabilities and limitations of people, but also influenced by wider elements in the environment and work system [ 11 ]. There is also significant evidence to show that organisational factors, like institutional policies, workforce training, and regulatory frameworks play a large role in whether a technology is selected, implemented and ultimately used [ 12 ].

Given the importance of HOF in successful adoption of technologies, and the realisation of benefits following health technology implementation, including planetary health benefits, this rapid integrative review sought to examine the extent to which HOFs are considered in current research on health technology and planetary health. We set out to review the current literature, identify key themes, and highlight areas for further study.

## 2. Methods

### 2.1. Study Design

A rapid integrative review was conducted using a structured approach to synthesize existing literature. A rapid review was chosen as a form of knowledge synthesis because it accelerates the process of conducting a traditional systematic review through streamlining or omitting specific methods to produce evidence for stakeholders in a resource-efficient manner [ 13 ]. The review followed the Population, Concept, and Context (PCC) framework to define the scope of the study and guide the selection of relevant literature [ 14 ].

### 2.2. Search Strategy

A literature search was conducted using Scopus and PubMed to identify relevant studies. The search strategy included a combination of keywords and controlled vocabulary terms related to health technologies and planetary health. Boolean operators, truncation, and filters were applied to refine the search results. A publication date filter was applied to refine the search results exclusively to the studies published in the last year (2024). The search terms used appear in Supplementary Table 1 .

### 2.3. Study Selection

A total of 463 studies were initially retrieved. The identified studies were imported into Covidence, a systematic review management tool, for screening and selection [ 15 ]. After removing 98 duplicate records, 365 unique studies remained for title and abstract screening. Titles and abstracts were independently screened by two reviewers (VT, KJ or MB) and any conflicts were resolved by a third reviewer. Following this stage, 26 studies were selected for full-text review to assess their eligibility. In total, 14 studies met the inclusion criteria and were included in the final data extraction phase. Figure 1 provides a PRISMA flowchart of the screening process.

**Figure 1.**
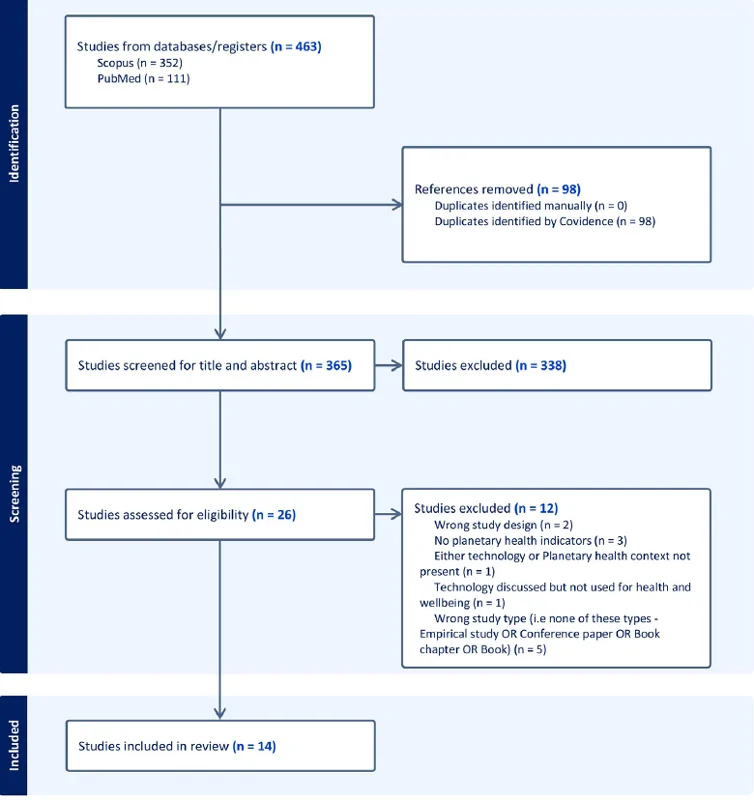
PRISMA flowchart for the screening process.

### 2.4. Eligibility Criteria

Studies were selected based on predefined inclusion and exclusion criteria, and criteria were determined using the PCC framework. The population criteria included all geographical regions, all age groups, all ethnicities and all settings. The concept criteria included studies investigating health technology, including virtual care. The context criteria included planetary health, global warming and climate change. Only empirical studies (journal and conference papers) published in English in 2024 were included in this study. Further details on the search strategy, keywords, and inclusion/exclusion criteria are available in the Supplementary Table 1 .

### 2.5. Data Extraction and Synthesis

Relevant data from the included studies were extracted using a standardized data extraction form ( Supplementary Table 2 ). Information such as study design, population characteristics, key findings, and context were recorded. The researchers then noted the papers that included findings or discussion related to HOFs. The findings were synthesized narratively to provide an integrative summary of the available evidence. That is, findings and relationships within and between studies are explained using words, not quantified.

## 3. Results

Data from 14 studies were extracted and key insights are described below. It was particularly noteworthy that no study considered human or organisational factors.

### 3.1. Geographical Distribution

Most of the studies in this review were conducted in the United States of America (USA) (n = 8) with remaining studies each based in Germany, India, Canada, the Netherlands, the United Kingdom (UK) and New Zealand.

### 3.2. Study Designs and Sample Sizes

There was limited diversity in the adopted study designs. All the studies used a retrospective observational study design with four studies also employing a Life Cycle Assessment process. As a result, all the studies utilised a quantitative methodology to estimate the impact on human and planetary health. No qualitative or mixed-methods approaches were used. Most studies provided insights from relatively large samples (NMin = 81, NMax = 251832). This could be attributed to the quantitative observational nature of the methodology employed by the studies.

### 3.3. Factors Investigated

A visual representation of the key outcomes measured (carbon dioxide [CO2] emissions, travel distance, etc.) is provided in Figure 2 .

**Figure 2.**
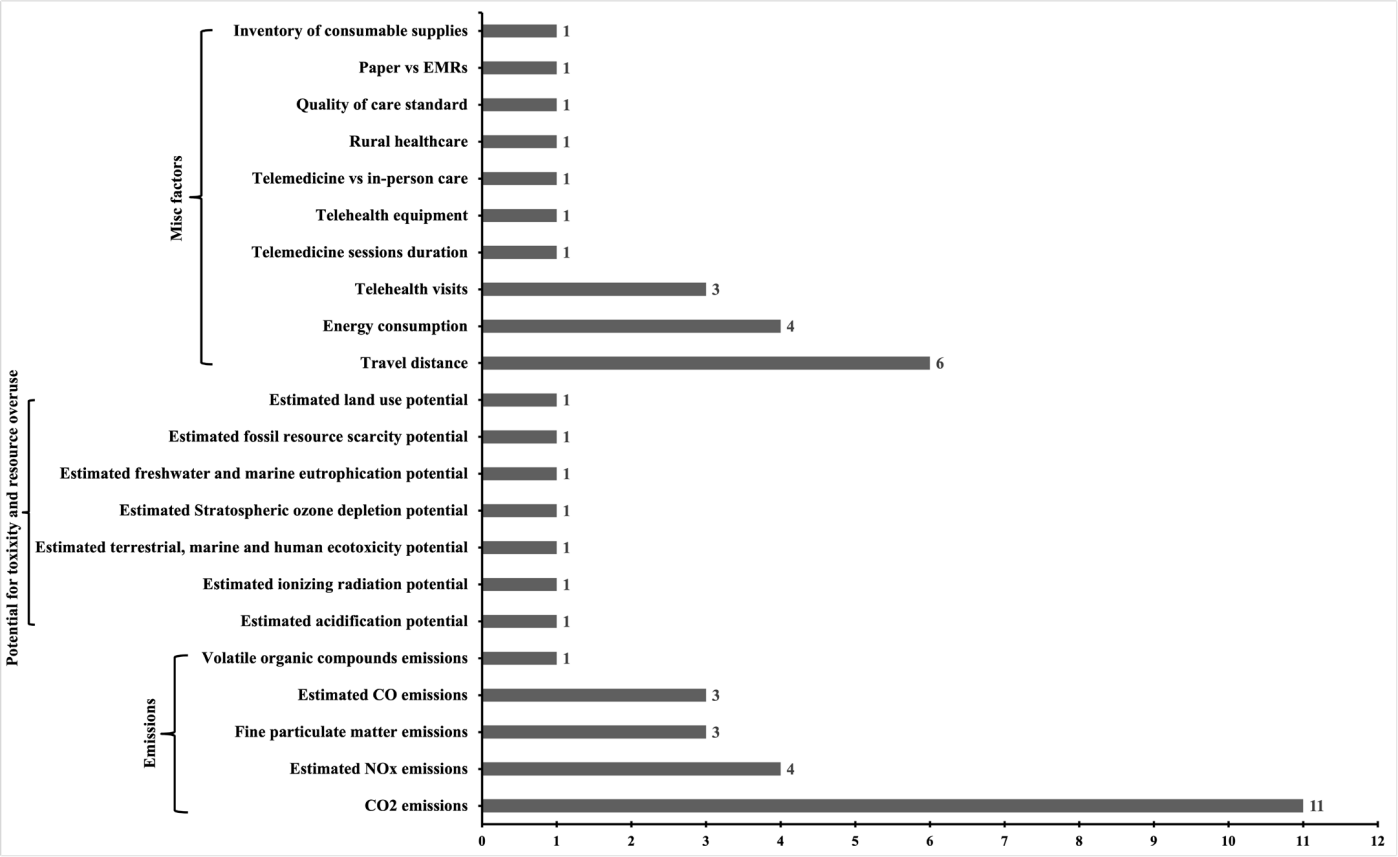
Bar chart highlighting the most frequently investigated factors across studies.

As shown in Figure 2 , estimated greenhouse gas emissions were the most frequently examined outcomes in these studies. Some of the other most frequently studied factors were travel distance, energy consumption and telehealth visits. Other crucial aspects like potential for toxicity, pollutant emissions (carbon monoxide [CO], Nitrogen Oxides [NOx]), and health technology infrastructure were less studied.

### 3.4. Types of Health Technologies and their Impact on Planetary Health

Health technologies used in the studies included virtual video consultations (n=11), remote patient monitoring and communication systems (n=2) and Electronic Medical Records (EMRs, n=1). Eleven studies in this review reported that virtual or tele-consultations reduced emissions by minimising patient travel and lower fuel consumption ( Supplementary Table 2 ). This reduction was observed across various healthcare settings, including cardiology, dermatology, oncology, urology, maternal-foetal medicine, and rural medical centres, where the adoption of telehealth services consistently demonstrated a net positive environmental impact [ 16 17 18 19 20 21 ]. Additionally, a study that evaluated home monitoring for conditions such as cystic fibrosis and elderly care found this use of health technologies contributed to a reduced healthcare-related carbon footprint as it also decreased the need for travel, therefore reducing emissions [ 27 ].

However, not all health technologies were reported to be environmentally beneficial. One study in this review estimated an increase in CO _2_ emissions due to energy consumption, material use, and disposal related to the use of EMR systems [ 23 ]. Another study demonstrated the negative impact of using digital communication and remote patient monitoring systems. Electronic equipment builds and setup requires rare metal mining and servers require a continuous supply of electricity. This resulted in higher electricity consumption, fossil resource scarcity potential, and environmental concerns related to mining such as ionizing radiation, stratospheric ozone depletion, acidification, and ecotoxicity [ 28 ]. Mining metals for health technologies were also found to impose additional burdens on land use and freshwater/marine ecosystems [ 28 ]. Finally, one study presented a balanced assessment of implementing virtual consultations and suggested that while digital healthcare infrastructure has environmental costs, the overall net effect of telemedicine remained beneficial due to the substantial reduction in travel-related emissions [ 24 ].

## 4. Discussion

Our rapid integrative review demonstrated there was research being conducted on the intersection of health technology and environmental concerns, however, there has been no consideration for HOFs, despite the large role HOFs play in technology design, adoption, implementation and sustainability. Current studies also had a reasonably narrow definition of what an environmental outcome was. Based on our findings the following key themes emerged.

### 4.1. Geographical Context

The geographical distribution of the studies in this review highlighted a significant concentration of research in the USA (57%), with only a small number of studies from other countries, including Germany, India, Canada, the Netherlands, the UK, and New Zealand. This geographic bias suggests that findings may not be fully generalizable to other regions globally, particularly those with different healthcare infrastructure, telehealth adoption rates, or socio-political contexts. For example, the adoption of health technologies in developing countries is often limited by infrastructure and maintenance costs and health and digital literacy rates, likely making telehealth a less common mode of service delivery and reducing its potential environmental benefits [ 30 ].

An underexplored area was telehealth accessibility, which varies significantly between urban and rural communities [ 31 ]. This in turn influences the extent to which telehealth can reduce travel distances and associated CO2 emissions. Rurality also directly affects the distance travelled by patients from their homes to the nearest medical centre as well as the mode of transport used [ 32 ]. Future research should explore telehealth adoption in underrepresented regions, with a focus on the HOFs specific to these contexts and evaluate the impact of rurality on a technology's effectiveness to mitigate environmental footprints.

### 4.2. Methodology

All the studies included in this review employed a quantitative retrospective observational approach, primarily focusing on estimated travel distances and associated CO2 emissions. While this methodology provides valuable insights on one aspect of environmental impact, it lacks an in-depth exploration of patient and staff perspectives on planetary health and health technology. The absence of qualitative and mixed methods approaches limits understanding of the HOFs influencing health technologies adoption and sustainability. Future research should incorporate diverse methodologies to capture the lived experiences, attitudes, behavioural responses of healthcare providers and patients, and any unintended or overlooked impacts of health technologies.

There is also a need for prospective quantitative studies in this area of research. This study design would allow us to better measure exact outcomes (such as distances and fuel), record multiple environmental outcome measures to analyse the broader impacts of health technologies in a specific setting or use case, and would allow for the capture of longitudinal health and environmental data.

### 4.3. Outcome Measures and Estimates

The studies predominantly measured CO2 emissions through proxy estimated travel distances, and in some cases, additional pollutants such as particulate matter (PM), CO, and NOx. However, these estimates were based on assumptions regarding patient travel distances and modes of transport, which introduce likely inaccuracies. Most studies calculated CO2 emissions by estimating travel distance using home addresses and assuming common modes of transport, often relying on averages rather than real-world data. The variability in fuel efficiency, car types (petrol, diesel, or electric), and alternative transport methods (public transport, cycling, or walking) were rarely considered in these calculations. This highlights the need for improved methodologies incorporating real-world data collection to validate these estimates. Furthermore, it would be worth examining the providers travel distances and frequencies especially in high impact areas such as in rural contexts.

The mode of transport used for healthcare visits is a critical factor in determining the environmental impact of travel-related emissions. However, the reviewed studies largely assumed that cars were the primary mode of transport, overlooking variations by country, region, and socio-economic context. For example, in urban European settings, public transport or cycling may be more common, whereas in rural North America, private car use is predominant (33). Additionally, studies often overlooked the rising adoption of electric vehicles, which significantly alters the CO2 emission estimates. Future research should refine emission calculations by incorporating local transport data and patient-reported travel behaviours.

### 4.4. Health Technologies and Environmental Costs beyond Travel

While most studies focused on the reduction of CO2 emissions through decreased patient travel with telehealth, few considered the environmental impact of digital technology itself. Telehealth services rely on electronic devices, which require mining of rare earth minerals and constant electricity usage for server maintenance and water for cooling servers [ 28 ]. The planetary cost of electronic waste, server infrastructure, and increased electricity consumption due to prolonged device usage is an understudied area. A comprehensive environmental assessment of telehealth taking a whole systems approach should include these factors to provide a more holistic understanding of its sustainability impact.

There is also a need to investigate health technology consumption and its environmental impact both at a system or organisation level and at the individual or patient level. For example, while health technology clearly decreases greenhouse gas emissions by reducing patient travel, its financial impact on a health organisation (who must invest in the hardware and cost associated with energy for software and infrastructure) must also be considered.

### 4.5. Gaps in Literature and Future Directions

We found limited investigation of the role of HOFs in impact of technologies on planetary health. This was despite Human Factors and Ergonomics being recognised as a discipline which contributes to achieving the sustainability development goal of “ensuring healthy lives and promoting wellbeing” via improving access to essential healthcare services, and the ergonomic design of healthcare facilities, tools and workflows [ 34 ]. The recent studies identified in this review did not acknowledge the importance of effective or optimal design of technologies and overlooked the perspectives of patients, healthcare providers and institutions in adopting and sustaining health technologies. Understanding how organizations implement, monitor, and report on sustainable healthcare practices is crucial for integrating health technology into planetary health initiatives. We know very little about whether knowledge of deteriorating planetary health would impact a provider or patient's decision to use a technology. Given the critical role that acceptance and use of technology plays in achieving expected benefits of technology [ 35 36 ], understanding if and how climate impacts are considered is critical to advancing scale up of health technologies, and reducing the negative impacts of technology on the environment such as energy use, e-waste and rare earth metal mining costs. It is also relatively unknown how health technologies can help deliver healthcare in situations that arise due to environmental changes such as wildfires and floods. Furthermore, adoption of technology and the impact of deteriorating planetary health on humans is not equitably levied. Developing nations, especially those surrounded by oceans are less likely to benefit from health technologies despite being at a higher risk of deteriorating environmental impact.

Estimated CO2 emissions and travel distance were the two most frequently used outcomes identified in this review. While there were studies that also investigated other pollutant emissions such as CO, NOx, fine particulate matter and energy use, our review highlighted that there are many understudied areas where health technologies may have an impact on the environment. Although this review has shown that health technologies can have a positive impact on planetary health, the current literature is heavily skewed toward travel-related emissions while neglecting to take a system-wide approach to technology design and in evaluating technology's overall environmental impact. The predominance of retrospective observational studies and quantitative methodologies leaves a gap in qualitative research exploring patient and provider perspectives on sustainability.

### 4.6. Encouraging Sustainable Health Technologies Adoption

To enhance the design and adoption of sustainable health technologies, future research should focus on incentivizing organizational participation and developing transparent monitoring and reporting frameworks. Policymakers and healthcare institutions should consider integrating sustainability assessments into health technology implementation strategies. Future advancements in technologies (i.e., increased capabilities) are likely to influence planetary impacts, and interdisciplinary collaborations between environmental scientists, healthcare providers, and technology developers could lead to more sustainable health technology innovations.

## 5. Conclusion

In conclusion, while health technologies offer promising results for reducing travel-related emissions, their broader environmental impact requires further investigation as does understanding the role that human and organisational factors play in technology design, adoption, implementation and maintenance. Addressing the identified research gaps will be essential for advancing sustainable healthcare practices that positively support planetary health. Finally, it would be prudent to consider the adverse effects of technology on the environment during large scale implementation and adoption of health technology.

### Limitations

Due to the rapid nature of this review, and the limited scope of the search to the most recently published studies, key publications published prior to 2024 would have been missed in this integrative review. Furthermore, the concepts of health technologies and planetary health are diverse and can include factors such as natural disasters, which were not included in this review. Our search strategy was constructed with the assistance of a University of Sydney librarian, but some search terms may have been missed from this review.
