# Supplementary material for: Health Technology and the Role of Human and Organisational Factors in Planetary Health: a Rapid Integrative Review of Current Literature
Source: Yearb Med Inform. 2026 Nov 3;34(1):241–9. doi: 10.1055/s-0046-1816625 (PMC13730676; doi:10.1055/s-0046-1816625)
Supplement: Supplementary file 1 [file 10-1055-s-0046-1816625-styagi.pdf]

Human Factors and Organizational Issues: Appendix

Supplementry Table 1. Keywords, search strategy and inclusion and exclusion criteria

Keywords for Concept	Keywords for Context
Digital health, Health information technology, Medical informatics, Health informatics, Telemedicine, Health Technology, eHealth, mHealth, Mobile health, Virtual hospital, Patient remote monitoring, Electronic medical record, Electronic health record, Telehealth, Virtual care, Computerised decision support, Clinical information system, Physician order entry system, Medical order entry system, Electronic prescribing	Planetary health, One health, Ecohealth, Environmental health, Health ecology, Health adaptation, Green health, Health resilience, Climate change, Greenhouse effect, Global warming, Extreme hot weather, Extreme cold weather, Sea level rise, Heat wave, Wildfires, Forest fire, fires, Fire season, Pollution
**Search terms and strategy**	
("Digital health" OR "Health information technology" OR "Medical informatics" OR "Health informatics" OR "Telemedicine" OR "Health Technolog*" OR "eHealth" OR "mHealth" OR "Mobile Health" OR "Virtual hospital*" OR "Patient remote monitoring" OR "Electronic medical record*" OR "Electronic health record*" OR "Telehealth" OR "Virtual care" OR "Computerised decision support" OR "Clinical information system*" OR "Physician order entry system*" OR "Medical order entry system*" OR "Electronic prescribing")<br><br>AND<br><br>("Planetary health" OR "One health" OR "Ecohealth" OR "Environmental health" OR "Health ecology" OR "Health adaptation" OR "Green health" OR "Health resilience" OR "Climate change" OR "Greenhouse effect" OR "Global warming" OR "Extreme*weather" OR "Sea level rise" OR "Heat wave" OR "Wildfire*" OR "Forest fire*" OR "Fire season" OR "Pollution")	
**Inclusion criteria**	
Search within	Article title, Abstract, Keywords
Year	2024-2025
Language	English
Publication types	Journal article, Conference paper
Publication stage	Published, In press
Study type	Quantitative, Qualitative, Mixed methods, Speculative/Simulation
**Exclusion criteria**	
Concept	Technology not used for health and wellbeing
Context	Studies with either technology or planetary health indicators but not both<br>Studies with no planetary health indicators

Supplementary Table 2. Summary of study characteristics

First author, year, country	Sample characteristics	Study design and Context	Factors investigated	Ref
Cummins<br>2024<br>USA	N=79,904 sessions<br><br>% Females = NR<br><br>Each session had<br>one patient &<br>one provider<br><br><400 miles geodesic distance<br><br>Calls less than 5 mins were excluded	Design: Retrospective Quantitative Observational Study of Telemedicine sessions<br><br>With estimates of CO2 emissions attributable to telemedicine in the US using national stats and geography<br><br>Travel distance was estimated using Google Distance Matrix Application Programming Interface for car travel (based on assumption that 84.8% of travel was by car consistent with American Community Survey Data on adult commuting patterns)<br><br>CO2 emissions per mile assuming an average fuel economy of 22 miles per gallon.	CO2 emissions<br>Telemedicine sessions duration<br>Geographical distance of patient from provider<br>Geographical distribution of Telemedicine sessions and CO2 emissions by county in the US	[15]
Finkelstein et al 2024<br>USA	N = 3,846 (before Covid)<br>N = 307,273 (after Covid)<br><br>% Females = 52%<br><br>Video visits = 99.9%<br>Telephone visits = 0.1%	Retrospective Observational study at single Quaternary-care children's hospital<br><br>Virtual consultation services<br><br>Estimated travel distance	Patient travel distance<br>Gasoline consumption<br>CO2 emissions<br>Fine particulate matter emissions<br>Hospital energy use	[16]
Grabski et al 2024<br>USA	N = 20845<br><br>% Female = 42%<br><br>Age = 8 years (median)	Retrospective Observational study of Electronic medical record system at an outpatient children's hospital centre<br><br>Rural area	Number of telemedicine visits<br>Estimated travel distance<br>CO2 emissions	[17]
Gruber et al 2024<br>Germany	N = 821<br><br>Travel by car/taxi = 81.7%<br><br>Teleconsultation mode = Video	Life Cycle Assessment using prospective surveys<br><br>Perioperative anesthesia teleconsultations at a hospital	Exact distance travelled<br>Mode of transport<br>Estimated CO2 emissions	[18]
Gunn 2024<br>USA	N = 9,942 patients<br>N = 14,828 sessions	Retrospective Observational Study at a Cardiology department at a hospital<br><br>Estimated travel distance using Home addresses of patients	Number of Telehealth visits<br>Estimated travel distance<br>Estimated CO2<br>Estimated CO<br>Estimated NOx<br>Estimated particulate matter	[19]
Hantel 2024<br>USA	N = 123,890<br><br>% Female = 59.7%<br><br>Age = 59 years (median)	Retrospective Observational study with Population based cohort study and counterfactual analyses using life cycle assessment<br><br>At a cancer institute<br><br>An in-person care period vs a telemedicine period	Estimated CO2 emissions<br>CO2 emissions between in-person vs. telemedicine sessions<br>Estimated Travel distance using home addresses	[20]
Hefferman et al 2024<br>Canada	N = simulated for whole population (Table 1)	Retrospective Observational study with modelling cohort study design at a children's hospital<br><br>Virtual visits	Estimated travel distance using home addresses<br>Estimated CO2 emissions	[21]

Kwon et al 2024<br>India	N = 1 centre	Retrospective Observational Study with Life cycle assessment and Impact assessment method at an eye care facility<br><br>Impact of paper record keeping vs Electronic medical record keeping	Estimated CO2 emissions<br>using : Inventory of consumable supplies; EMR capital outlay and disposal; Energy consumption	[22]
Jones et al 2024<br>New Zealand	N = 484<br>% female = 64%<br>Age = 55 years (mean)<br>60% from urban<br>36% semi-rural<br>4% rural	Retrospective observational study at a dermatology department<br><br>Virtual consultations	Estimated CO2 emissions for the use of computers, monitors, lighting and heating<br>Estimated CO2 emissions for travel taking into account Car, bus and non-vehicle forms of travel e.g. walking and cycling	[23]
Li Valverde et al<br>2024<br>USA	N = 2,454<br>Age = 28.7 (mean)	Retrospective observational study in maternal-fetal-medicine clinics<br><br>Tele consultations	Estimated travel distance using google maps<br>Estimated CO2 emissions, CO, NO, and volatile organic compounds	[24]
Madison et al<br>2024<br>USA	N = 251,832	Retrospective observational study in a purely rural setting in a medical centre in three time periods – pre-pandemic, early and late pandemic<br><br>Telehealth visits	Estimated travel distance<br><br>Estimated CO2 emissions assuming standard passenger vehicle	[25]
Oppelaar et al<br>2024<br>The Netherlands	N = 81<br>% female = 60%<br>Age = 26 (median)	Retrospective multicentre observational study in 5 cystic fibrosis centres<br><br>Telehealth - People with home monitoring of lung function with portable spirometers and symptoms with questionnaires	Estimated travel distance using google maps<br>Quality of care standard<br>Estimated CO2 emissions<br>Healthcare consumption patterns	[26]
Tarpani et al<br>2024<br>The UK	N = 3 centres<br><br>N = 1105, 45, 38 residents	Retrospective observational study using Life cycle assessment In elderly living centres<br><br>Telehealth equipment – Living hub, Door entry, Video desk phone, Handset, Pendant, Base unit, A/Cs<br><br>Tele communication devices	Estimated electricity consumption<br>Estimated CO2 emissions<br>Estimated fossil resource scarcity potential<br>Estimated Stratospheric ozone depletion potential<br>Estimated Nox<br>Estimated Fine particulate matter<br>Estimated ionising radiation potential<br>Estimated acidification potential<br>Estimated freshwater and marine eutrophication potential<br>Estimated terrestrial, marine and human ecotoxicity potential<br>Estimated land use potential	[27]
Wurtz et al<br>2024<br>USA	N = 126	Retrospective observational analysis in an outpatient urology clinic<br><br>Telehealth visits	Estimated distance using google maps for car travel<br>Estimated CO2 emissions	[28]

Copyright

© 2025. The Author(s). This is an open access article published by Thieme under the terms of the Creative Commons Attribution License, permitting unrestricted use, distribution, and reproduction so long as the original work is properly cited. https://creativecommons.org/licenses/by/4.0/
